# A genome-wide investigation of insidious uveitis in Appaloosa horses

**DOI:** 10.1186/s12864-025-12099-3

**Published:** 2025-10-09

**Authors:** N. B. Kingsley, L. Sandmeyer, A. Dwyer, C. D. Langefeld, R. J. McMullen, M. McCue, M. Lassaline, R. R. Bellone

**Affiliations:** 1 Veterinary Genetics Laboratory, School of Veterinary Medicine, University of California - Davis, Davis, CA 95616 USA; 2 Department of Population Health and Reproduction, School of Veterinary Medicine, University of California – Davis, Davis, CA 95616 USA; 3 Department of Small Animal Clinical Sciences, Western College of Veterinary Medicine, University of Saskatchewan, Saskatoon, Saskatchewan S7N 5B4 Canada; 4 Genesee Valley Equine Clinic, LLC, Scottsville, NY 14546 USA; 5 Department of Biostatistics and Data Science, Division of Public Health Sciences, Wake Forest University School of Medicine, Winston-Salem, NC 27101 USA; 6 Center for Precision Medicine, Wake Forest University School of Medicine, Winston-Salem, NC 27101 USA; 7 Equine Department, Vetsuisse Faculty, University of Zurich, Zurich, Switzerland; 8 Veterinary Population Medicine Department, College of Veterinary Medicine, University of Minnesota, St. Paul, MN 55108 USA; 9 School of Veterinary Medicine, University of Pennsylvania, Philadelphia, PA 19104 USA

**Keywords:** ERU, GWAS, Inflammation, Equine, Moon blindness, Periodic ophthalmia, Panuveitis

## Abstract

**Background:**

Equine recurrent uveitis (ERU), an inflammatory eye disease, is the leading cause of blindness among horses. Insidious uveitis, a form of ERU, is especially pervasive within the Appaloosa breed and is highly heritable (h^2^ = 0.68-1.0). To date only one risk locus, leopard complex (*LP*), has been identified, and it explained 0.16–0.33 of the heritability estimate, suggesting that insidious uveitis is a complex genetic disease within the Appaloosa horse breed with multiple unknown predisposing loci.

**Results:**

A genome-wide association study (GWAS) using relatedness, *LP* genotype, sex, and age as covariates was performed on a sample of 96 Appaloosas (36 cases and 60 controls) and identified a 9.7 Kb region of association on ECA X (chrX:14528106–14537812) as significantly associated (*P* = 2.11 × 10^−8^). Sex stratification followed by meta-analysis provided additional support for the association on ECA X (*P* = 1.35 × 10^−8^). A logistic regression model was performed to test for epistasis between *LP* and the locus on ECA X, and the results did not support an interaction between the two loci. In the second phase of the study, single-nucleotide variants (SNVs) were identified in the region on ECA X by whole genome sequencing (WGS) of 18 horses from the GWAS (9 cases and 9 controls). Five reference markers from the GWAS, two previously associated coat color loci (LP and PATN1), and 102 SNVs were further evaluated in a combined dataset of 157 horses (70 cases and 87 controls, including the original 96 horses from the GWAS). Using logistic regression, none of the SNVs identified from the WGS analysis were significantly associated with phenotype; however, *LP* and the top three SNP markers from ECA X (ECA X: 14.5 Mb) were significantly associated in the larger dataset (P_LP_ = 2.34 × 10^−6^ and P_X_ = 4.06 × 10^−5^).

**Conclusion:**

In addition to the *LP* locus, our investigation identified a locus on chromosome X with a significant association to insidious uveitis in Appaloosas. Replication testing in an independent cohort is necessary to determine if this locus is indeed a causal risk locus.

**Supplementary Information:**

The online version contains supplementary material available at 10.1186/s12864-025-12099-3.

## Background

Equine recurrent uveitis (ERU) is a group of inflammatory diseases affecting critical tissues of the inner eye. As the leading cause of blindness for horses, ERU can have a detrimental impact on a horse’s quality of life. Previous investigations found that the majority of affected horses could not return to pre-disease performance roles, and a large proportion of cases (15–53%) were euthanized during these studies as a direct result of ERU [1, 2]. Furthermore, diagnostic and treatment costs per case commonly reached $1,000–3,000 and $3,000–5,000, respectively [2]. Such financial burdens and impact on the horse’s quality of life make this ocular disease a major concern for the horse industry.

ERU is characterized by inflammation within the vascular uveal tract, and it has been categorized based on location where inflammation predominates, such as posterior (behind lens) versus anterior (in front of lens), as well as the manner of inflammation, such as classic (episodic inflammation) versus insidious (continuous inflammation) [3–5]. In particular, the insidious form of ERU is characterized by persistent, low-grade inflammation and usually presents with subtle or even no outward signs of intraocular inflammation, making this form of the disease difficult to recognize before irreversible damage has occurred [3, 6].

**Fig. 1:**
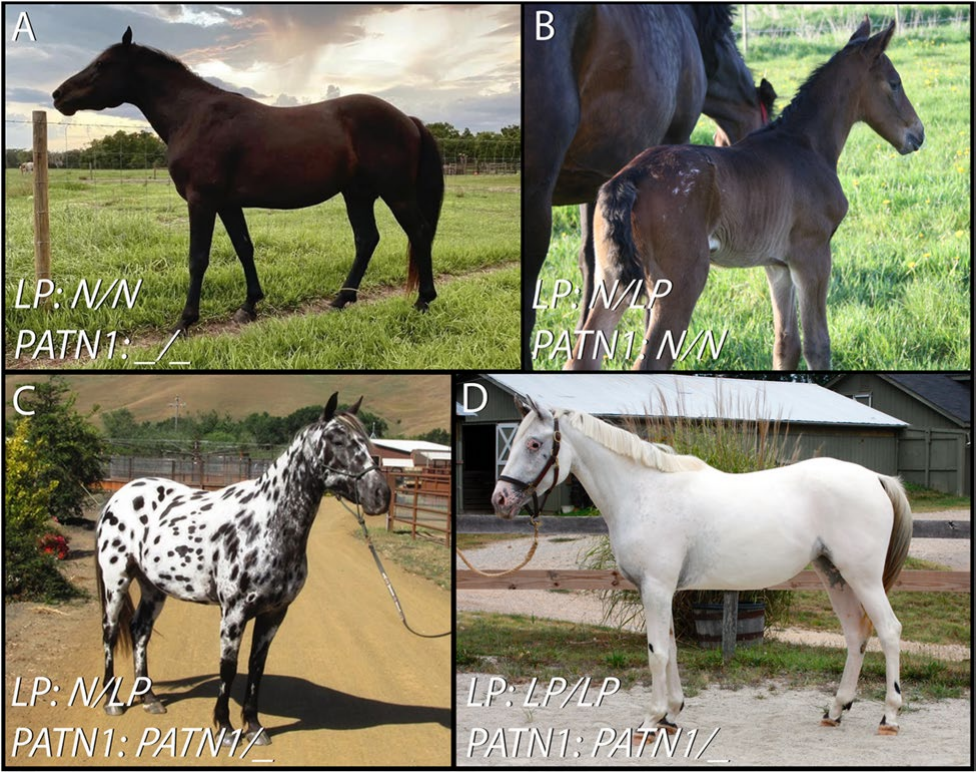
An Illustration of Leopard Complex Spotting Coat Patterns (LP) and the Alleles Involved. **A** A horse without any copies of *LP* (the genotype at this locus is *N/N)*. These horses are considered “true solids” as they do not display any phenotypic characteristics of the leopard complex spotting pattern, regardless of *PATN1* genotype. **B** A heterozygous *LP* horse without any *PATN1* alleles showing minimal white patterning over the rump. **C** Heterozygous *LP* horse with extensive white patterning due to the *PATN1* locus. The horse’s coat contains pigmented spots within the unpigmented region, which is characteristic of *N/LP* horses. **D** A horse with two copies of *LP* that also displays extensive white patterning due to the combination of genotypes at the *LP* and *PATN1* loci. *LP* homozygous horses usually have a minimal number of pigmented spots within the white patterned area. Photographs courtesy of (A) Chelsea Thornton, (B) Dr. Sanna Hèden, (C) Cheryl Woods, and (D) Martha Mitchell

Predisposition for insidious uveitis among Appaloosa horses and a high heritability estimate (h^2^ = 0.68-1.0) provide evidence of a strong genetic component underlying insidious uveitis in this breed [6–8]. Interestingly, Appaloosa horses have been selected for a particular coat pattern known as leopard complex spotting pattern that has been previously associated with ocular disease. The leopard complex spotting phenotype is denoted as LP (without italics), and it is characterized by white patterning starting over the hindquarters that can vary in size from minimal white patterning to an entirely unpigmented coat (Fig. 1). The coat pattern is an incomplete dominant trait caused by a long terminal repeat (LTR) insertion in an intron of the *Transient Receptor Potential Cation M1 Channel* (*TRPM1*) gene [9, 10]. The insertion allele is responsible for producing a large variety of patterns with varying levels of unpigmented coat. In accordance with how the genotype is commonly reported in genetic tests, the insertion allele is known as *LP* (in italics), and the wildtype allele is denoted as *N*. Modifier loci are thought to be responsible for the phenotypic variation observed. In addition to influencing the coat pattern, *LP* is also known to disrupt the function of the ON-bipolar cell signaling pathway that is necessary for proper transmission of light detection from the rod cells of the retina to the ON-bipolar cells along the neural pathway to the brain [9, 11, 12]. As a result, horses homozygous for the insertion have a disorder known as Congenital Stationary Night Blindness (CSNB) [10].

Previous research using a candidate gene approach found that insidious uveitis in a sample of Appaloosas was associated with two markers in the set of immune genes known as the major histocompatibility complex (MHC) and one marker in the 11th intron of *TRPM1*, tagging *LP* [7]. Subsequent testing on the SNP70 BeadChip genotyping array provided further support for the association with *TRPM1* but did not support the association to the MHC [13]. It was suspected, however, that evaluation on an array with a higher marker density, such as the Axiom Equine 670 K Genotyping Array, may be necessary to properly tag the notoriously variable MHC. Given the association between this region and ERU in German Warmbloods and similar associations between human autoimmune uveitis and the equivalent human region (ELA), the MHC has remained an important area of investigation for insidious uveitis risk factors [14–17].

Two investigations further supported the association of the *LP* allele with disease risk among Appaloosas [13, 18], and data from one of these studies suggested an association between insidious uveitis and *PATN1*, an allele at the *Ring Finger And WD Repeat Domain 3* (*RFWD3*) gene [13]. *PATN1* was previously identified as a modifier of *LP* leading to more extensive white patterning [19]. The other investigation found that a larger amount of white patterning at birth was associated with increased risk for insidious uveitis, although *PATN1* was not tested directly in the second study [18]. Both *LP* and *PATN1* are of particular interest given the selection for the LP phenotype in the Appaloosa and other related breeds, and together these investigations indicate that *LP* and potentially *PATN1* or closely linked variants, are involved in the development of insidious uveitis. However, further evaluation of *PATN1* is necessary to determine if the association with insidious uveitis can be replicated. A model with age, *LP*, and *PATN1* genotypes was not sufficient to fully explain the distribution of this disease among Appaloosas (AUC = 0.83), suggesting that additional genetic loci may be involved in the development of insidious uveitis in this breed [13]. Based on previous research in horses and humans, as well as the immunogenic nature of the disease, we hypothesize that a large proportion of risk for insidious uveitis in Appaloosas can be explained by multiple genetic loci, including *LP* and *PATN1*.

## Results

### Identifying regions of interest by association testing

**Fig. 2:**
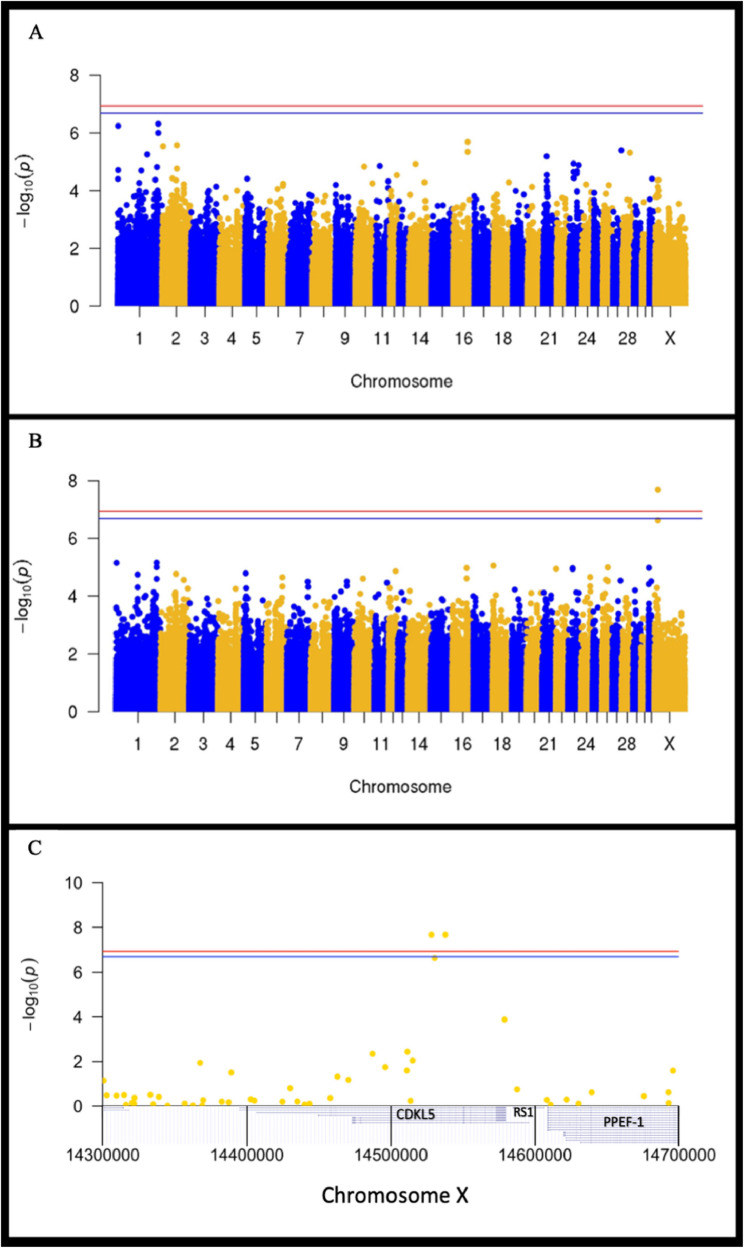
GWAS for insidious uveitis in a sample of 96 Appaloosa horses. Manhattan plots from association testing using Genome-wide Efficient Mixed Model Association (GEMMA). X-axes represent genomic position, and Y-axes represent statistical association. Red horizontal lines represent a Bonferroni significance threshold (α_bonf_. = 1.17 × 10^−7^), and blue horizontal lines represent a modified Bonferroni adjusted significance threshold (α_mod.bonf_. = 2.05 × 10^−7^) based on the number of independent markers. **A** Model 1 - mixed linear model with a kinship matrix, sex, and age as covariates. **B** Model 2 - mixed linear model with a kinship matrix, sex, age, and *LP* genotypes as covariates. **C** Zoomed-in Manhattan plot of the most associated region from Model 2, overlying the EquCab3.0 gene positions from the UCSC genome browser

Under a mixed linear model (MLM) from Genome-wide Efficient Mixed Model Association (GEMMA) software with a relationship matrix, sex, and age as covariates (called Model 1), no regions reached genome-wide significance, although two regions on ECA 1 (ECA 1: 171.8 Mb, *P* = 4.81 × 10^−7^ and ECA 1: 2.5 Mb, *P* = 5.74 × 10^−7^) approached significance, based on multiple testing correction for the number of independent tests (α_mod.bonf_. = 2.05 × 10^−7^) (Fig. 2A; Table 1). By including *LP* genotype as an additional covariate, a second MLM analysis (Model 2) did not support either locus on ECA 1 but instead identified a 9.7 Kb region reaching genome-wide significance on the X chromosome (ECA X: 14.5 Mb, *P* = 2.11 × 10^−8^) (Fig. 2B). Figure 2C provides a locus zoom plot of the chromosome X region. The genomic inflation factors for the two MLM analyses were 1.05 and 1.04, respectively.

**Table 1:** Wald test (MLM) *p*-values for the top five SNP markers from GWAS analyses of 96 horses (36 cases and 60 controls) using model 1 and model 2

				Model 1^b^			Model 2^c^		
SNP	Chr	Position	Allele	OR^a^	95% CI^a^	*P*-value	OR^a^	95% CI^a^	*P*-value
AX-104,215,907	1	2,453,754	T	9.00	2.73–29.65	5.74 × 10^−7^	9.61	2.52 - 36.71	7.17 × 10^−6^
AX-104,449,150	1	171,786,187	G	7.05	2.89 - 17.23	4.81 × 10^−7^	7.95	2.82 - 22.39	7.08 × 10^−6^
AX-104,277,711	X	14,528,106	C	3.07	1.72 - 5.48	1.53 × 10^−4^	11.77	3.89 - 35.67	2.11 × 10^−8d^
AX-104,167,796	X	14,530,368	A	3.84	1.94 - 7.61	4.33 × 10^−5^	9.08	3.27 - 25.24	2.35 × 10^−7^
AX-104,228,393	X	14,537,812	T	3.13	1.76 - 5.57	9.88 × 10^−5^	11.68	3.86 - 35.38	2.11 × 10^−8d^

^a^OR: odds ratio for additive genetic model, 95% CI: 95% confidence interval for odds ratio

^b^Model 1: GEMMA mixed linear model with a kinship matrix, age, and sex as covariates

^c^Model 2: GEMMA mixed linear model with a kinship matrix, age, sex, and LP genotypes as covariates

^d^Denotes significance using a modified Bonferroni adjusted threshold (αmod.bonf. = 2.05 × 10−7) based on the number of independent markers

To formally test for a chromosome X association and for differential effects by sex, a sex stratified analysis with subsequent meta-analysis was completed, and the three associated SNP markers on the X chromosome continued to be significantly associated with insidious uveitis in the meta-analysis (*P* = 1.35 × 10^−8^). The effect size (i.e. odds ratios) were relatively comparable in female and male Appaloosas, and there was no evidence of a sex-ECA X interaction (*P* > 0.05).

### Investigating region of interest by whole genome sequencing

The region evaluated for variants on ECA X included 150 Kb up and downstream of the associated locus (ECA X: 14.37–14.68 Mb). Of the 1,552 variants identified in WGS data from 18 horses (9 cases and 9 controls), only five variants had an allele frequency difference of 50% or greater between the cases and controls used for sequencing. Based on filtering by genotype frequency difference of 0.3 or greater between cases (*n *= 9) and controls (*n *= 9), 130 SNVs from ECA X were identified for further investigation by genotyping in a larger cohort of samples (*n* = 157) (Table S1). Furthermore, only one coding variant was identified in the region, but it was not predicted to have a deleterious impact on protein function (synonymous variant). Four variants were identified within the boundaries of histone mark ChIP-Seq peaks, suggesting a potential role in gene regulation [20, 21]. To better assess associations in the larger cohort, seven additional markers (*LP*, *PATN1*, two markers on ECA 1 approaching genome-wide significance in Model 1, and three significant markers on ECA X identified in Model 2) were also evaluated. *PATN1* was included in this analysis to compare with previous association studies [13, 18]. After quality filtering, 109 variants and 157 samples (70 cases and 87 controls) remained for further analysis. Using a logistic regression model from GEMMA with sex and age as covariates (Model A), none of the 102 WGS SNVs investigated were perfectly concordant with phenotype in the full dataset of 157 horses, and only an association with the *LP* locus reached significance after multiple testing correction with a Bonferroni adjusted threshold (*LP*, P = 2.34 × 10^−6^) (Table 2). After adding *LP* genotype as another covariate (Model B), the three GWAS SNP markers from ECA X reached significance (X_14530368, P = 4.06 × 10^−5^; X_14537812, P = 4.13 × 10^−5^; X_14528106, P = 4.14 × 10^−5^). These three markers are tightly linked (all pairwise D’ = 1 and r^2^ > 0.4; Table 3). *PATN1* was not significantly associated with insidious uveitis in any analysis, and none of the chromosome X SNVs identified by WGS showed a significant association with disease phenotype, even after inclusion of *LP* as a covariate (Table S2); albeit the analyses were only able to detect larger effects. Post-hoc power analysis indicates that the sample size of 157 horses was sufficient (1-β = 0.8) for identifying moderately common loci (0.3 ≤ AF ≤ 0.4) with odds ratios ≥ 5 or very common loci (0.6 ≤ AF ≤ 0.85) with an odds ratio ≥ 15. *PATN1* had an allele frequency of 0.2 in the larger dataset and an anticipated effect size below OR = 5.

**Table 2:** Investigating *LP, PATN1*, and five SNP markers by genotyping in a larger dataset of 157 Appaloosas (70 cases and 87 controls). *P*-values for associations between insidious uveitis and *LP*, *PATN1*, and five SNP markers originally identified in models 1 and 2

			Model A^b^			Model B^a^		
SNP	Chr	Position	OR^a^	95% CI^a^	*P*-value	OR^a^	95% CI^a^	*P*-value
*LP*	1	109,211,964	6.90	3.27–14.52	2.34 × 10^−6d^	n/a	n/a	n/a
*PATN1*	3	24,352,525	2.01	1.12 - 3.59	0.52	1.27	0.66 - 2.42	0.65
AX-104,215,907	1	2,453,754	5.57	2.14 - 14.48	0.083	4.55	1.57 - 13.13	0.037
AX-104,449,150	1	171,786,187	3.31	1.76 - 6.25	0.102	3.56	1.74 - 7.29	4.22 × 10^−3^
AX-104,277,711	X	14,528,106	2.27	1.46 - 3.54	0.71	2.83	1.67 - 4.81	4.14 × 10^−5d^
AX-104,167,796	X	14,530,368	2.36	1.54 - 3.61	0.072	2.84	1.71 - 4.73	4.06 × 10^−5d^
AX-104,228,393	X	14,537,812	2.29	1.48 - 3.56	0.61	2.83	1.67 - 4.80	4.13 × 10^−5d^

^a^*OR* Odds ratio for additive genetic model, *95% CI *95% confidence interval for odds ratio

^b^Model A: logistic regression model with age and sex as covariates

^c^Model B: logistic regression model with age, sex, and LP genotypes as covariates

^d^Denotes significance using a strict Bonferroni corrected threshold (αbonf. = 4.59 × 10−4)

**Table 3:** Linkage disequilibrium between three SNP markers. Using PLINK, pairwise r^2^ was calculated for the three significantly associated SNP markers from model 2

Chr	Position	SNP	AX-104,277,711	AX-104,167,796	AX-104,228,393
X	14,528,106	AX-104,277,711		0.465	1
X	14,530,368	AX-104,167,796	0.465		0.469
X	14,537,812	AX-104,228,393	1	0.469	

A receiver operating characteristic (ROC) curve was used to evaluate logistic models with and without the X locus (AX-104277711) as an independent variable in predicting disease status. The Area Under the Curve (AUC) for Model C (phenotype ~ *LP* genotype + sex + age) is 0.78, indicating that 78% of the variation in disease risk is explained by this model. The AUC for Model D (phenotype ~ *LP* genotype + sex + age + marker AX-104277711 genotype) is 0.83, and thus, approximately 5% of the variation in disease risk within Our dataset can be explained by the associated region on ECA X. The curve for model D has an optimal threshold value of 0.53, specificity (rate of true negatives) of 0.89, and sensitivity (rate of true positives) of 0.63.

## Discussion

Previous investigations in Appaloosas characterized the *LP* insertion as a pleiotropic locus, conferring both the defining coat pattern of the Appaloosa breed and the ocular condition known as CSNB [9, 10, 12]. As seen in previous investigations, the *LP* locus was significantly associated with insidious uveitis when evaluated within the larger dataset in this study [13, 18]. The *LP* insertion contains an early poly-adenylation signal that prevents transcription of the entire *TRPM1* gene within retinal tissues [10]. Investigations in humans have also found novel mutations in *TRPM1* associated with CSNB, often in conjunction with myopia and strabismus phenotypes similar to the clinical findings reported in horses [22, 23]. Molecular investigations indicate that absence of functional TRPM1 is the cellular mechanism leading to an inability to see in scotopic conditions [18, 24–28]. Evaluation of *TRPM1* expression in homozygous (*LP/LP*) and heterozygous (*N/LP*) horses indicate that both experience a decrease relative to *N/N* individuals (*LP/LP* = 0.0005 and *N/LP* = 0.31), yet heterozygotes produce enough wild-type TRPM1 for normal light detection response after dark adaptation [12].

Although *LP/LP* horses have been identified as having the highest risk for developing insidious uveitis compared to *N/N* (OR = 19), heterozygous individuals are also affected by insidious uveitis [13, 18]. Furthermore, *LP* genotype has been found to explain part, but not all, of the phenotypic distribution of insidious uveitis among Appaloosas [8, 13]. While it is valuable to use *LP* genotype as a risk factor for management decisions (i.e. *LP/LP* horses should be evaluated by a veterinary ophthalmologist more frequently), it is not clear if the association with insidious uveitis is the result of linkage or causality, and further interrogation of the region by haplotype analysis is needed to refine the locus. Additional investigations are also needed to characterize the cellular ramifications of the *LP* insertion to determine what role it may play in the etiology of insidious uveitis. In humans, TRPM1 has been connected to a paraneoplastic autoimmune disease of the eye known as melanoma associated retinopathy, in which overexpression of *TRPM1* in the melanocyte tumor likely promotes development of autoantibodies targeting TRPM1 [29]. Since this disease indicates that an autoimmune response can target the TRPM1 protein, it raises the question about what role the aberrant *TRPM1* allele may play in promoting ocular inflammation in Appaloosa horses with insidious uveitis.

Evaluation of *PATN1* genotypes in this expanded cohort of 157 horses did not identify an association with disease status; however, post-hoc power analysis suggests that this study was not sufficiently powered for detecting loci with effect sizes smaller than OR = 5. Thus, *PATN1* may play a minor role in the genetic contribution to insidious uveitis risk as suggested by previous association studies, but a larger cohort may be needed to identify an effect from this locus or other minor effect loci in cases of insidious uveitis [13, 18].

To account for the known risk associated with the *LP* locus, *LP* genotype was used as a covariate to identify additional putative risk loci. Using a MLM with kinship, sex, and age as covariates (Model 1), two regions on ECA 1 approached genome-wide significance; however, the associations on ECA 1 were no longer significant after accounting for *LP* genotype in the GWAS (Model 2). Additionally, the two markers on ECA 1 were not as concordant with insidious uveitis phenotype as *LP* when genotyped in the larger cohort (*n* = 157). Therefore, it is suspected that these loci were only tagging the *LP* insertion, and further interrogation of SNVs from this ECA 1 region was not performed.

When accounting for relatedness, *LP* genotype, sex, and age in the MLM analysis (Model 2), three SNP markers tagging a 9.7 Kb region on ECA X reached genome-wide significance, and the same markers continued to be significantly associated with disease in a sex-stratified analysis. Although we cannot completely exclude the possibility that the association is the result of sampling bias due to non-random sampling, the repeated associations while correcting for sex indicate that the association is not driven by an imbalanced sex ratio in the dataset. When comparing AUC values for logistic regression models with and without a term for the associated X locus (Models C and D), the X locus accounts for approximately 5% of variation in phenotype within our dataset that is not explained by sex, age, or *LP* genotype. Previous research indicates that the heritability of insidious uveitis among Appaloosa horses is high (REML 95% CI, *h*^2^ = 0.68–1.0) with *LP* contributing 16–33% of the variation in the phenotype [8]. Our results suggest that the associated region on ECA X has the potential to explain a smaller portion than the *LP* locus, but these two loci together with age and sex can explain up to 83% of the variation in our dataset. It is important to recognize that the association identified in this study on ECA X does not warrant diagnostic genetic testing at this time as functional interpretations cannot be made from GWAS results, and additional work is needed to assess the replicability of the association in an independent dataset.

None of the 102 SNVs investigated from the associated X locus and 150 Kb flanking regions was perfectly concordant with phenotype in the full dataset of 157 horses. Further testing of the WGS variants with logistic regression did not support a significant association with any of the 102 SNVs, even when *LP* was used as a covariate. Yet, the three SNP markers on ECA X from Model 2 remained significantly associated with insidious uveitis in the larger dataset when *LP* was included as a covariate (Model B). There are several possible explanations for the continued association of these X chromosome markers. First, this region may be a false positive, and the region is incidentally concordant with the disease phenotype in the sample sets used in this study. To rule out this possibility, another association study with an independent sample as large or larger than this study (*n* = 157) of affected and unaffected Appaloosa horses is needed. Alternatively, the region could be associated with insidious uveitis due to an unknown confounder rather than a true causal variant. Third, these markers may be tagging an uncharacterized mutation, such as a structural variant, that was not investigated in this study. More WGS and functional work are needed to investigate this hypothesis should replication testing further support the association to this region.

The three significantly associated X chromosome SNP markers in this study are all located within introns of the *cyclin dependent kinase Like 5* (*CDKL5*) gene and are in high linkage disequilibrium. This gene is associated with neurologic disorders in humans and is, therefore, not an obvious functional candidate gene for insidious uveitis [30]. *Protein phosphatase with EF hand domain-1* (*PPEF-1*), located 30 Kb downstream of our GWAS hit, is also an unlikely functional candidate gene. It has limited expression within human ocular tissues, and knockout experiments of *PPEF-1* and its paralog *PPEF-2* did not lead to retinal degeneration in mice [31, 32]. One synonymous coding variant of *PPEF-1* was identified in the WGS cohort, but the mutation was not concordant with insidious uveitis in the larger dataset (*P* = 0.21). A plausible candidate gene, retinoschisin 1 (*RS1*), is located between *CDKL5* and *PPEF-1*, and mutations in the gene are associated with a retinal delaminating disease known as retinoschisis in humans [33]. The protein is necessary for proper retinal structure by mediating cell adhesion and interactions between photoreceptors and bipolar cells [34]. Humans with dysfunctional *RS1* generally experience progressive visual loss with retinal lesions and retinoschisis, as well as retinal detachment or degeneration [33]. Although uveitis in humans has not been associated with mutations in *RS1*, investigations of knockout mice demonstrated that TRPM1 positioning within the membranes of bipolar cells is altered in the absence of the RS1 protein, suggesting a possible functional link between *TRPM1* and *RS1* in normal ocular tissue [35]. Since the associated region on ECA X was only significant in our investigations when *LP* genotype was considered, we suspected that the pathogenic impact of the X locus may depend on the presence of the *LP* allele. An interaction analysis using logistic regression with a centered cross-product interaction term did not support the hypothesis that *LP* is epistatic to the X locus; however, it should be noted that the statistical power for an interaction between loci in this sample is modest for plausible effect sizes. Although no single-nucleotide coding variants were identified in *RS1* based on WGS of 18 horses, functional investigations to explore the expression of *RS1*, as well as *PPEF-1*, and *CDKL5*, in affected and unaffected ocular tissues may be warranted if the chromosome X locus is supported by replication testing.

Similar to other investigations of diseases in domesticated animals, the sample size in this study limited the ability to detect loci with small effect size. Here, the modeling carefully considered covariates to not induce collinearity and adjusted for relatedness to maximize the robustness of the models. Future investigations should aim to utilize larger cohorts or share data to enable more powerful meta-analyses by combining the available data. Additionally, unknown environmental risk factors may have affected our analyses. Whenever feasible, cohabitating cases and controls were used to mitigate the effect of environmental variation within the analyses; however, perfectly paired cases and controls were not available from every farm. Further work is needed to determine specific environmental factors contributing to insidious uveitis pathology to build more robust risk models and better understand disease progression within the Appaloosa breed. Despite these limitations, our investigation suggests that future research on insidious uveitis would benefit from further assessing the functional role of the *LP* locus in the progression of this disease.

## Conclusion

Our investigation provided additional support of the *LP* locus as a risk factor of insidious uveitis in Appaloosa horses and identified a locus on ECA X as another putative risk factor. Additional work is needed to test the replicability of the association on ECA X in an independent population of Appaloosa horses and other LP breeds to understand the potential role of this locus in the etiology of insidious uveitis.

## Methods

The aim of the study was to investigate additional loci potentially impacting insidious uveitis disease predisposition in the Appaloosa breed using case-control GWAS and WGS analyses.

### Sample collection

All protocols were approved by the Institutional Animal Care and Use Committee at the University of California, Davis (18851, 20699, and 22466) or by the Animal Care Committee at the University of Saskatchewan (20110053). All horses received a complete ocular examination from a Doctor of Veterinary Medicine (DVM), a diplomate or honorary member of the American College of Veterinary Ophthalmologist (ACVO), or a diplomate of the European College of Veterinary Surgeons (ECVS). The exams included neuro-ophthalmic, transilluminator, slit-lamp biomicroscopic, indirect ophthalmoscopic, and tonometry assessments. Horses experiencing active uveitis (aqueous or vitreous flare, conjunctivitis, miosis, blepharospasm, epiphora, and photophobia) with evidence of past inflammation received a diagnosis of insidious uveitis. Additionally, a diagnosis of insidious uveitis was given to horses with sequelae indicative of long-term inflammation within the eye, including lens luxation, synechiae, mature cataracts, phthisis bulbi, glaucoma, iris atrophy and color changes, corneal neovascularization, and retinal detachment or degeneration. Samples of blood and/or hair follicles were collected from each examined horse, and DNA was isolated using the Gentra Puregene DNA Isolation kit (Qiagen Inc.), following previously validated protocols [36].

An initial sample set of 96 phenotyped Appaloosa horses from Rockwell et al. (2020) and Kingsley et al. (2022) was used in this study [8, 13]. These genotyping data are available in the Open Science Framework repository (MAP and PED files): https://osf.io/v6s4f/. Given the replicated association to *LP*, these horses were selected for inclusion to evaluate other variants for association with insidious uveitis; thus, the sample contains a high proportion of *LP* heterozygous horses (*n* = 58/96) among both the cases (*n* = 21/36) and controls (*n* = 37/60). For variant investigation, an additional cohort of 61 phenotyped Appaloosas (not tested previously) was combined with the dataset of 96 horses for a total sample set of 157 animals (70 cases and 87 controls). Given the progressive nature of this disease, control horses had to be at least eight years of age with no clinical evidence of disease at the time of the examination and no history of eye disease. The *LP* and *PATN1* loci were assayed for all horses by the UC Davis Veterinary Genetics Laboratory diagnostic testing services.

### Genome-wide association study of insidious uveitis in 96 Appaloosas

After quality control filtering of the genotype data (minor allele frequency < 0.05, genotype call rate < 0.90, and sample call rate < 0.90), 96 horses (36 cases and 60 controls) and 426,343 markers remained for the analysis. GWAS was performed using MLMs from GEMMA software [37, 38]. GEMMA reduces the influence of genomic inflation by accounting for relatedness and population structure using a genetic relationship matrix as a covariate and performs a Wald statistical test of association. Model 1 is a mixed Linear model with a kinship matrix, sex, and age as covariates. Model 2 is a mixed linear model with a kinship matrix, sex, age, and *LP* genotypes as covariates. To correct for multiple testing in the GWAS, strict Bonferroni and modified Bonferroni adjusted thresholds were applied (α_bonf_. = 1.17 × 10^−7^ and α_mod.bonf_. = 2.05 × 10^−7^). The modified Bonferroni adjusted threshold is a multiple testing correction method that is based on the effective number of independent tests (i.e. unlinked genetic markers), and it was calculated using the Genetic Type 1 Error Calculator (GEC) software [39]. While simulation studies have not been performed specifically in horses to validate GEC, this approach for calculating the number of independent tests has been successfully utilized in several studies in horses and many investigations in other animal and plant species [40–63]. PLINK was used to calculate the pairwise D’ and r^2^ for the most associated markers [37].

To ensure that an uneven distribution of males and females did not lead to false positive associations in the GWAS, an analysis with stratification by sex was performed. Separate MLMs from GEMMA software were used to analyze the data from the males (*n* = 45, 21 cases and 24 controls) and the females (*n* = 51, 15 cases and 36 controls). Both models included relatedness, age, and *LP* genotype as covariates. The sex-stratified associations were then evaluated by meta-analysis with METAL software v.20,110,325, which converted the p-values into Z-scores for each individual analysis before combining them into a single weighted Z-score for each marker [64]. Additionally, logistic regression modeling in R v.4.1.3 was used to assess possible genetic interactions (epistasis) [65]. The interaction analysis coded female ECA X genotypes as 0 (homozygous reference), 1 (heterozygous), or 2 (homozygous alternate) and hemizygous male genotypes as 0 (reference) or 2 (alternate). Age and sex were used as covariates in the logistic model, and interaction terms were calculated as centered cross products.

### Whole genome sequencing of 18 Appaloosas

Whole genome sequencing of nine cases and nine controls from the GWAS dataset was performed to a target of at least 20X coverage per sample. Barcode-indexed sequencing libraries were generated from genomic DNA samples sheared on an E220 Focused Ultrasonicator (Covaris, Woburn, MA). For each sample, 37 ng sheared DNA were converted to sequencing libraries using the Kapa High Throughput Library Preparation Kit (Kapa Biosystems-Roche, Basel, Switzerland). The Libraries were amplified with 4 PCR cycles and analyzed with a Bioanalyzer 2100 instrument (Agilent, Santa Clara, CA), quantified by fluorometry on a Qubit instrument (LifeTechnologies, Carlsbad, CA), and combined in one pool at equimolar ratios. The pools were quantified by qPCR with a Kapa Library Quant kit (Kapa Biosystems-Roche, Basel, Switzerland), and the pool was sequenced on four lanes of Illumina NovaSeq 6000 (San Diego, CA) as paired-end 150 bp reads with a target insert size of 450 bp. Reads were trimmed and filtered for quality with HTStream v.1 software prior to alignment with BWA mem v0.7.16 [66, 67]. SAMtools v.1.11 was used to sort and consolidate files, and both Freebayes v. 1.3.1 and BCFtools v1.10.2 were used for variant calling [68, 69]. SNVs were characterized with SNPeff v.4.3 using the GenBank (GCA_002863925.1) annotation from NCBI, and SNVs were annotated for non-coding functional elements specific to equine tissues using publicly available histone mark ChIP-Seq peaks from the Functional Annotation of Animal Genomes (FAANG) Initiative (PRJEB35307 and PRJEB42315) [20, 21, 70, 71]. All variants within the region of interest (1,552 variants total) were filtered based on genotype frequency differences of at least 0.3 between cases and controls, and only variants identified by both variant callers were investigated further.

Genotyping of variants in a larger cohort of samples (*n* = 157 including the original GWAS cases, 111 cases and 139 controls) was performed on the Agena Bioscience MassARRAY system (San Diego, CA), and data were filtered using the same quality control parameters used in the GWAS (minor allele frequency < 0.05, genotype call rate < 0.90, and sample call rate < 0.90). Association testing of variants was performed using logistic regression from GEMMA [37, 38]. Model A is a logistic regression model with age and sex as covariates. Model B is a logistic regression model with age, sex, and *LP* genotypes as covariates. A strict Bonferroni adjusted threshold was used for multiple testing correction (α_bonf_. = 4.59 × 10^−4^). Post-hoc power analysis was performed with freely-available online software known as Genetic Power Calculator using an estimated prevalence of 0.25 and high linkage disequilibrium between marker and effect alleles [72]. Receiver operating characteristic (ROC) curves were calculated using Proc package from R for assessing logistic regression models with and without the genotypes from an associated SNP marker on ECA X (*AX-104277711)* [73]. Model C (phenotype ~ *LP* genotype + sex + age) and Model D (phenotype ~ *LP* genotype + sex + age + marker AX-104277711 genotype) were calculated using the glm function from R software (v. 4.5.1) [65].

## Supplementary Information

Supplementary Material 1.

Supplementary Material 2.

## Data Availability

The genotyping data are available in the Open Science Framework repository (MAP and PED files): [https://osf.io/v6s4f/]. The WGS data supporting the conclusions of this article are available on the European Nucleotide Archive (ENA) under the following project accession numbers: PRJEB88483 and PRJEB36380.
