# Supplementary material for: A genome-wide investigation of insidious uveitis in Appaloosa horses
Source: BMC Genomics. 2025 Oct 9;26:904. doi: 10.1186/s12864-025-12099-3 (PMC12513139; doi:10.1186/s12864-025-12099-3)
Supplement: Supplementary file 2 — Supplementary Material 2. [file 12864_2025_12099_MOESM2_ESM.docx]

**Supplementary Figure 1: GWAS for insidious uveitis in a sample of 96 Appaloosa horses.** PP plots of Wald test p-values with diagonal red line indicating perfect normality. (A) Observed p-values from a mixed linear model using GEMMA software including age and sex as covariates. The genomic inflation factor was 1.05. (B) Observed p-values from a mixed linear model using GEMMA software including age, sex, and LP genotype as covariates. The genomic inflation factor was 1.04.


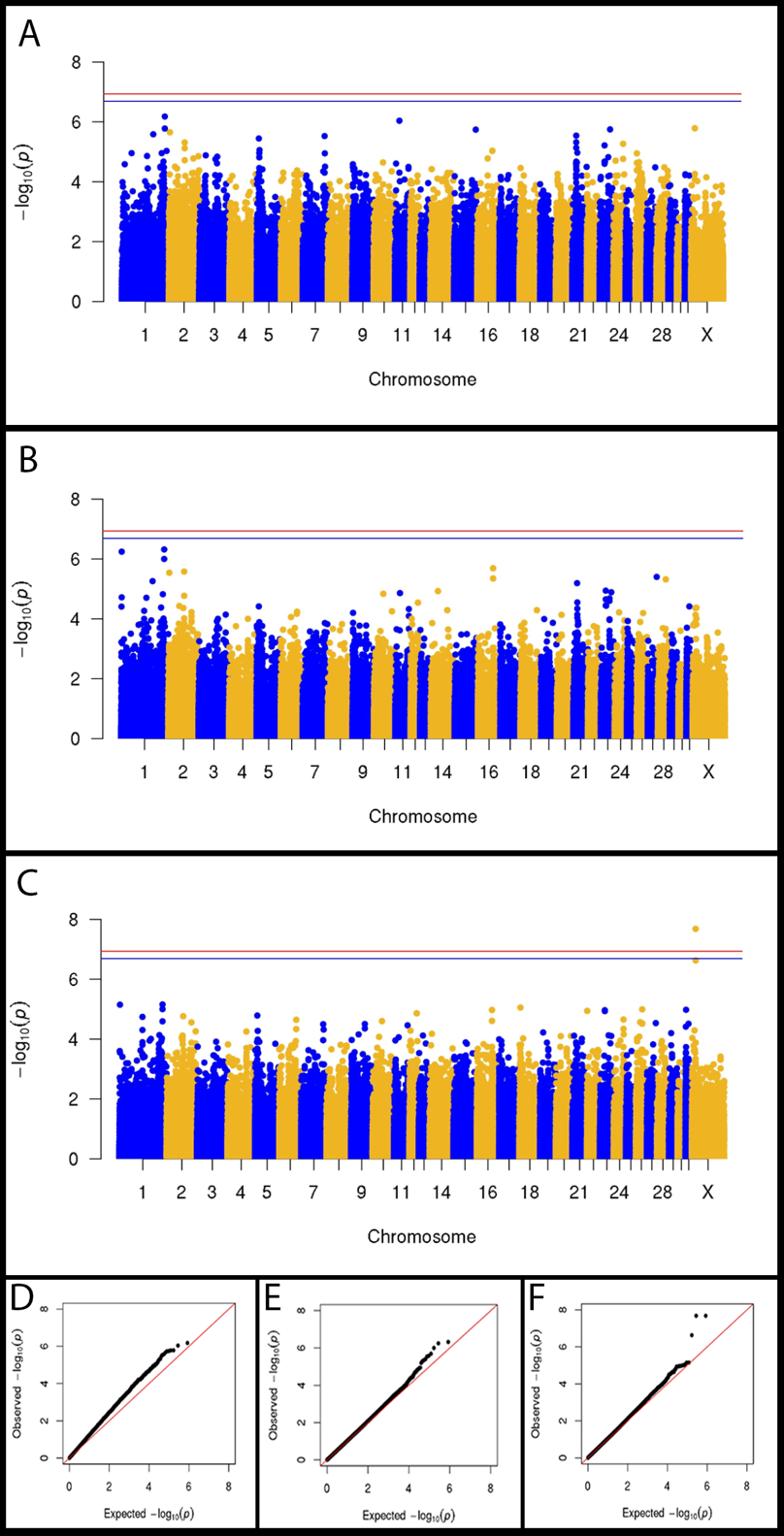


A

B

**Supplementary Table 1: Summary of 130 SNVs from ECA X identified through WGS of nine cases and nine controls.** The region evaluated for variants on ECA X included 150 Kb up and downstream of the associated locus (ECA X: 14.37-14.68 Mb). All variants identified in the region were filtered based on genotype frequency differences of at least 0.3 between cases and controls. Only the 130 variants identified by both variant callers were investigated further.

**Supplementary Table 2: Summary of association analysis for 102 SNVs 102 WGS SNVs investigated in the full dataset of 157 horses.** Of the 130 variants identified for further investigation by WGS, 109 variants remained after the data were filtered using the following quality control parameters (minor allele frequency < 0.05, genotype call rate < 0.90, and sample call rate < 0.90). A logistic regression model from GEMMA was used to assess these variants in a sample of 157 samples (70 cases and 87 controls).
